# Utilization of Olive Pomace in Green Synthesis of Selenium Nanoparticles: Physico-Chemical Characterization, Bioaccessibility and Biocompatibility

**DOI:** 10.3390/ijms23169128

**Published:** 2022-08-15

**Authors:** Emerik Galić, Kristina Radić, Nikolina Golub, Dubravka Vitali Čepo, Nikolina Kalčec, Ena Vrček, Tomislav Vinković

**Affiliations:** 1 Faculty of Agrobiotechnical Sciences Osijek, Josip Juraj Strossmayer University of Osijek, 31000 Osijek, Croatia; 2 Faculty of Pharmacy and Biochemistry, University of Zagreb, 10000 Zagreb, Croatia; 3 Institute for Medical Research and Occupational Health, 10000 Zagreb, Croatia

**Keywords:** selenium nanoparticles, olive pomace, polyphenols, bioaccessibility, biocompatibility

## Abstract

Olive pomace extract (OPE) was investigated as a potential surface modifier for the development of the green synthesis process of selenium nanoparticles (SeNPs). In order to evaluate them as potential nutraceuticals, the obtained nanosystems were characterized in terms of size distribution, shape, zeta potential, stability in different media, gastrointestinal bioaccessibility and biocompatibility. Systems with a unimodal size distribution of spherical particles were obtained, with average diameters ranging from 53.3 nm to 181.7 nm, depending on the type of coating agent used and the presence of OPE in the reaction mixture. The nanosystems were significantly affected by the gastrointestinal conditions. Bioaccessibility ranged from 33.57% to 56.93% and it was significantly increased by functionalization of with OPE. Biocompatibility was investigated in the HepG2 and Caco2 cell models, proving that they had significantly lower toxicity in comparison to sodium selenite. Significant differences were observed in cellular responses depending on the type of cells used, indicating differences in the mechanisms of toxicity induced by SeNPs. The obtained results provide new insight into the possibilities for the utilization of valuable food-waste extracts in the sustainable development of nanonutraceuticals.

## 1. Introduction

Selenium is an essential microelement in humans and animals and its role has been recognized in the nervous, thyroid, reproductive and immune systems [1,2]. Its sufficient intake is essential for the maintenance of cellular homeostasis, through the activity of selenoproteins and selenoenzymes [3,4]. Three groups of selenoenzymes are involved in the cellular antioxidative response: glutathione peroxidases (GPx), thioredoxin reductases (TrxR) and methionine sulfoxide reductase (MSR). Iodothyronine deiodinase (DIO) is involved in the metabolism of thyroid hormones in the thyroid gland, while selenoprotein P (SePP) functions as a carrier protein in human plasma. Additionally, selenoproteins are involved in calcium metabolism of the endoplasmic reticulum [5,6,7]. Special consideration is given to the selenoenzymes involved in the antioxidative response. The selenium atom, incorporated into the active site of enzymes, enables them to behave as strong reducing agents and neutralize ROS generated by cells’ oxidative metabolism. They are also involved in the regulation of cellular redox signaling [4,8]. The role of selenium as an antioxidant is indirectly exerted through the activity of selenoenzymes, and for this reason, it has been recognized as a promising candidate in anticancer research and the development of novel functional foods [6,9,10]. Selenium has a small therapeutic window with a low margin of dosage error. At excessive intake levels, it behaves as a prooxidant, which can lead to undesirable effects such as hair and nail loss or brittleness, lesions of the skin and nervous system, nausea, diarrhea, skin rashes, mottled teeth, fatigue, irritability, and nervous system abnormalities [11]. Its toxicity is primarily dependent on the dose, type of selenium compound and redox state of selenium, where organic forms seem to be less toxic in comparison to inorganic selenium salts [12].

Selenium is also available in the form of nanoparticles (NPs), which are generally characterized by unique features such as a high surface-to-volume ratio, solubility, and multi-functionality [13,14,15]. The application of nanotechnology in the development of nutraceuticals offers numerous advantages such as increased bioavailability, promoting the controlled-release and targeted delivery of encapsulated bioactive natural compounds, which leads to an increase in their biological efficacy [16].

Recent research on SeNPs has shown that they exert stronger biological activity and lower toxicity compared to standard forms [10,17,18]. Nano selenium has been investigated as a food supplement, an anticancer therapeutic and a drug carrier [15,19,20,21,22]. Various methods can be utilized to generate NPs, such as physical and chemical methods or biological synthesis [23], and the application of biological materials in the synthesis of NPs has been of particular interest. Specifically, microorganisms and plant extracts have shown the most promising results [24,25,26]. It has been reported that biogenic NPs have desired characteristics while being environmentally safe [27,28]. SeNps are currently being widely used as antimicrobial agents, growth promoters, crop biofortifiers and nutraceuticals in agriculture [29,30], while their biomedical applications are still limited. However, due to their exceptional catalytic, photoreactive, biocidal, anticancer and antioxidant properties, they are being investigated for use in antimicrobial coatings, nutritional supplements, nanotherapeutics, diagnostics, medical devices and other applications [31].

In the design of nutraceutical delivery systems, it must be taken into account that the formulation must have adequate chemical–physical properties, and lately, particular attention has been given to the development and application of benign-by-design processes devoted to environmental sustainability for the recovery and utilization of bioactive compounds from food waste [32]. Food waste represents a large source of high-value components such as polyphenols, pectins, proteins, carbohydrates and various antioxidants [33]. Unfortunately, the majority of food and agro-industrial waste is not being exploited, which presents a major ecological problem [34]. Efforts to utilize valuable waste for the development of novel materials have been described [35,36,37]. Olive pomace, a valuable form of waste from olive oil production, is rich in antioxidants, especially polyphenolic compounds such as hydroxytyrosol, oleuropein and tyrosol [38,39,40]. Due to their conjugated systems, polyphenols exert direct antiradical and reductive activity by acting as electron or hydrogen donors, which, in turn, leads to free-radical scavenging [41,42]. Additionally, they can contribute to the activation of antioxidative enzymes, namely glutathione peroxidase, catalase and superoxide dismutase, and to the regulation of cellular redox signaling; additionally, they can act as heavy-metal chelators [42]. The overall result is the mitigation of cellular oxidative stress, which, in turn, has favorable effects on the prevention of cancer, neurological and cardiovascular disorders, and aging [43].

The major goal of this investigation was to develop the process of the green synthesis of novel SeNPs through the utilization of olive pomace extracts rich in polyphenolic compounds, and to investigate their bioaccessibility and biocompatibility using validated in vitro models. We hypothesized that olive pomace polyphenols might serve both as the reducing agents necessary for the reduction of selenite in the process of SeNP synthesis, and as functional stabilization agents that might improve the physico-chemical characteristics and bioaccessibility of SeNPs. An additional goal of the study was to expand the current knowledge of the potential utilization of biologically active extracts obtained from food waste in the context of the emerging importance of green chemistry and sustainability in the field of nutraceutical development.

## 2. Results and Discussion

### 2.1. Synthesis and Purification of SeNPs

Obtaining SeNPs through chemical reduction requires the presence of a selenium source (selenium salts, selenium oxides, selenium acids or amorphous selenium) as a reducing agent and ionic/steric stabilizer in the reaction mixture. Under adequate conditions (the correct concentration of reactants, temperature, duration of chemical reaction, stirring conditions, etc.) the formation of SeNPs occurs and can be monitored as the increase in absorbance at characteristic wavelengths. The absorption maximums of SeNP UV-VIS spectra depend on the particle diameter and are usually in the range of 300–800 nm [44].

As reviewed extensively in the work of Korde and co-workers (2020) [45], much attention has recently been paid to the development of plant-assisted methods of SeNP synthesis, whereby plant extracts rich in natural antioxidants, particularly polyphenols, are used as reducing agents. In our work, the standard chemical synthesis of SeNPs was modified via the addition of the polyphenol-rich extract of olive pomace. The olive pomace extract used in the synthesis process contained significant amounts of hydroxytyrosol, tyrosol and oleuropein (71.7, 23.5 and 31.9 mg/100 g, respectively) and had high reducing potential (237.6 mg gallic acid equivalents (GAE)/100 g). Therefore, it was expected to provide higher yields in comparison to the standard synthesis procedure. This was eventually confirmed, as is obvious from the data presented in Table 1.

In the development of the synthesis process, it is crucial to adequately purify the obtained NPs by removing the remaining reactants present in the reaction mixture in order to obtain discrete NP fractions with a narrow range of sizes or density of the particles. This improves the sample quality and enables meaningful discussion of the functional properties of SeNPs in relation to their physico-chemical characteristics.

**Figure 1:**
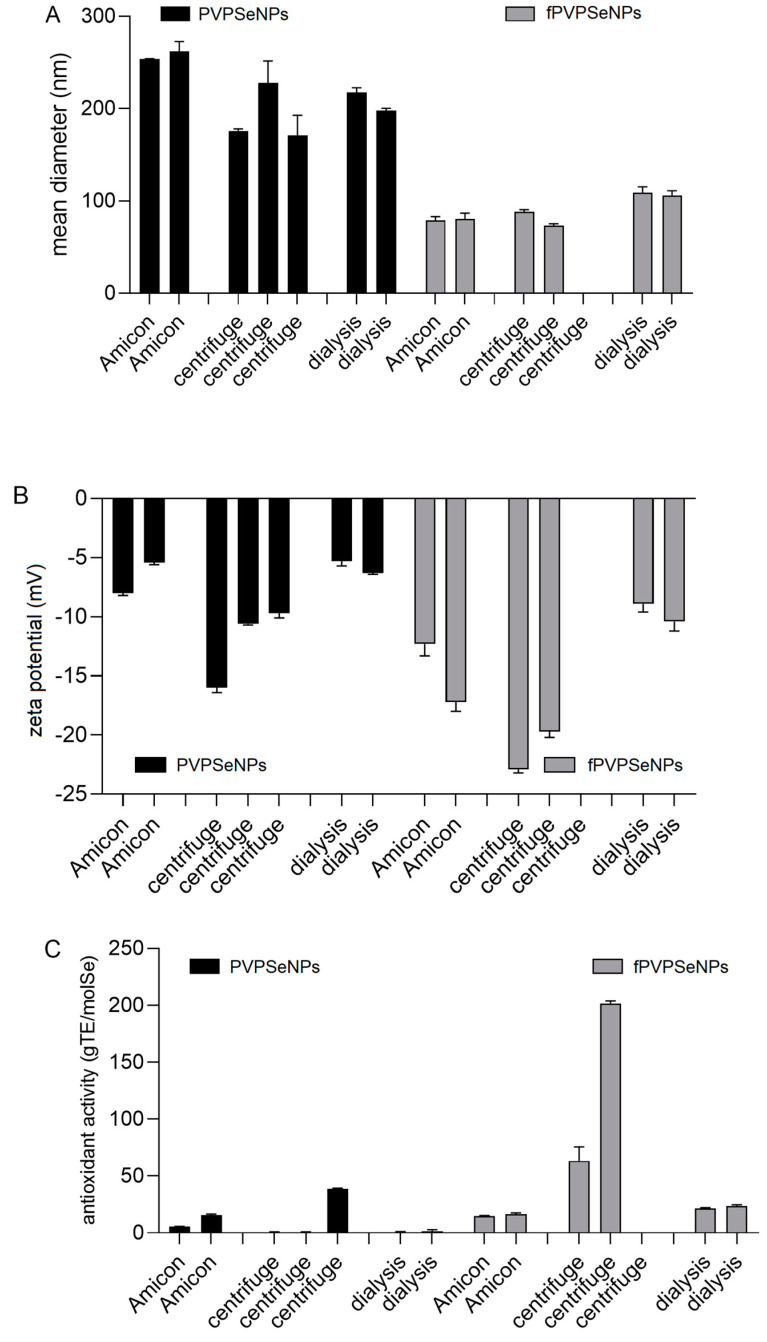
Impact of different separation techniques on mean hydrodynamic diameter (**A**), zeta potential (**B**) and Trolox-equivalent antioxidant capacity (TEAC) (**C**) of PVPSeNPs and fPVPSeNPs. (PVPSeNPs—selenium nanoparticles coated with polyvinylpyrrolidone; fPVPSeNPs—selenium nanoparticles coated with polyvinylpyrrolidone and functionalized with olive pomace extract). PVPSeNPs—selenium nanoparticles coated with polyvinylpyrrolidone; fPVPSeNPs—selenium nanoparticles coated with polyvinylpyrrolidone and functionalized with olive pomace extract. Multiple columns for same data sets indicate the number of replicates.

Several techniques have previously been employed to purify NPs and separate them by size and shape [46]. As presented in Figure 1, in our work, we investigated the impact of filtration, Amicon*^®^* filter-assisted filtration and dialysis on the size (Figure 1A), zeta potential (Figure 1B) and Trolox-equivalent antioxidant capacity (TEAC) (Figure 1C) of SeNPs coated with polyvinylpyrrolidone (PVPSeNPs), and on SeNPs coated with polyvinylpyrrolidone and functionalized with OPE (fPVPSeNPs).

An investigation of antioxidant activity was conducted in order to investigate whether the remaining reactants were successfully removed from the reaction mixture; namely, since PVPSeNPs do not scavenge free radicals (as established in the preliminary investigations), the absence of direct antioxidant activity can be considered as an indication of a successful separation/cleaning process.

**Figure 2:**
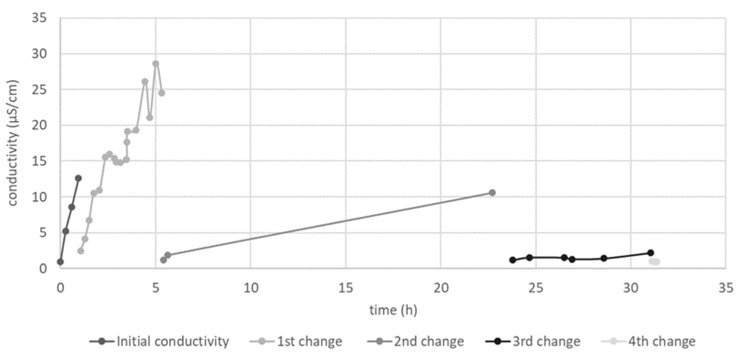
Optimization of the dialysis process for the purification of SeNPs.

As presented in Figure 1, the average size and zeta potential of the investigated nanosystems were significantly influenced by the applied separation technique. The NPs obtained via purification with Amicon*^®^* filters had the largest average diameter (>250 nm), while the smallest NPs were obtained via centrifugation. However, cleaning via centrifugation resulted in the highest variability of the obtained results and it was impossible to obtain satisfactory separation between SeNP precipitate and supernatant, resulting in huge losses of NPs during the removal of supernatant. Applying higher speeds to improve separation resulted in the formation of precipitates that were impossible to disperse. Figure 1c shows that in SeNPs purified via Amicon*^®^*-assisted filtration, and especially those obtained via centrifugation, showed direct antioxidant activity, probably due to the remaining L-ascorbic acid and/or OPE, indicating an incomplete purification process. On the other hand, SeNPs purified via dialysis did not show direct antioxidant activity, indicating complete purification. The necessary duration of dialysis was previously optimized by measuring the conductivity of the outer phase during the process (Figure 2). The conductivity measured before the start of dialysis was 0.99 μS/cm. Multiple changes in the dialysis buffer were made, in order to maintain the driving force of dialysis, after 1 h, 5 h, 23 h and 31 h. The highest value of conductivity was recorded after the first 5 h of dialysis (28.6 µS/cm). The conductivity values remained below 2 µS/cm for 24 h after the 3rd change in the dialysis buffer, initiating the end of the purification process.

### 2.2. Characterization and Investigation of Stability of SeNPs

**Figure 3:**
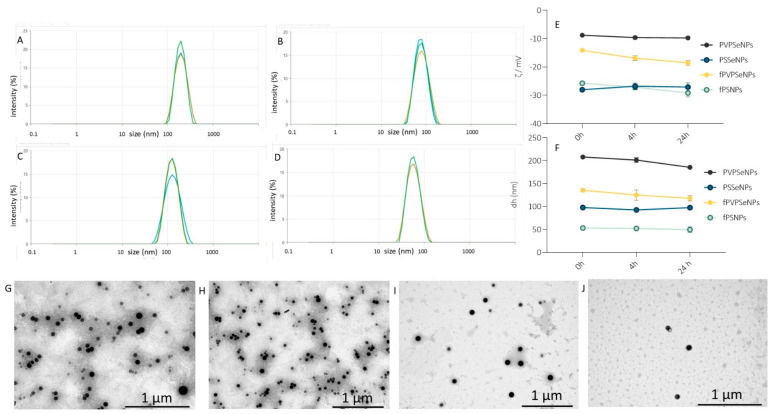
Size distribution by intensity. Stability and transmission electron microscopy (TEM) images of SeNPs: size distribution of PVPSeNP (**A**), PSSeNPs (**B**), fPVPSeNPs (**C**) and fPSSeNPs (**D**); 24h stability of zeta potential of NPs (**E**); 24h stability of an average diameter of NPs (**F**); TEM images of PVPSeNP (**G**), PSSeNPs (**H**), fPVPSeNPs (**I**) and fPSSeNPs (**J**). PVPSeNPs—selenium nanoparticles coated with polyvinylpyrrolidone; fPVPSeNPs—selenium nanoparticles coated with polyvinylpyrrolidone and functionalized with olive pomace extract; PSSeNPs—selenium nanoparticles coated with polysorbate; fPSSeNPs—selenium nanoparticles coated with polysorbate and functionalized with olive pomace extract.

As indicated by several recent studies, the main potential advantages of SeNPs in comparison to inorganic salts or organic forms of selenium (selenomethionine or selenocysteine) are higher bioavailability and reduced toxicity (i.e., a wider therapeutic range). Therefore, there is a growing interest in investigating SeNPs as a promising alternative to peroral application and food supplementation. The most crucial step for obtaining stabile nano-systems with suitable particle-size distribution is the selection of an adequate type and amount of encapsulation/stabilization agent. Based on the data obtained within our preliminary investigation, polyvinylpyrrolidone (PVP) and polysorbate Tween 20*^®^* (PS) were selected as stabilization agents to be combined with OPE, which served as a potential nanoparticle surface modifier. The obtained SeNPs were characterized in terms of size, shape, zeta potential and stability given that those characteristics are crucial for the prediction of the expected biological activity (Figure 3).

The transepithelial permeability of NPs is predominantly determined by their size. For example, crossing the mucus layer implies a particle size below 200 nm [47], and the efficiency of transcellular transport into the enterocyte is reversely correlated with the average diameter of the nanoparticle. For example, retinoic acid NPs showed a 3-fold increase in oral bioavailability when particle size was reduced from 328.8 nm to 89.3 nm [48]. The smaller nanostructured lipid carriers (NLC-100 nm) show higher uptake efficiency in the Caco-2 model (*p* < 0.05) as well as higher permeation ability in the Caco-2 cell monolayer (*p* < 0.01) compared with NLC-200 nm and NLC-300 nm [49]. Generally, satisfactory cellular uptake has been shown for particles with diameters lower than 150 nm [17].

Within a given geometric shape, a nanomaterial’s size is a strong determinant of its total cellular uptake. As presented in Figure 3, the obtained NPs were spherical in shape, and their shape was unaffected by functionalization with OPE. The average diameter of NPs ranged from 56.0 nm (SeNPs coated with PS and functionalized with OPE-fPSSeNPs) to 181.7 nm (PVPSeNPs). Generally, polysorbate-stabilized NPs (PSSeNPs) were smaller in comparison to PVPSeNPs, making them potentially more suitable for peroral application. According to the available data, a 50 nm diameter is optimal for increasing the rate of uptake and intracellular concentration in certain mammalian cells [50]. The average diameter of NPs was significantly decreased via functionalization with OPE and the obtained average diameters were well below 200 nm (107.6 nm and 56.0 nm, respectively), which is considered suitable for oral application in terms of achieving satisfactory bioavailability. The polydispersity indexes of the obtained NPs ranged from 0.11–0.18, pointing to narrow, unimodal particle-size distribution. Functionalization with OPE significantly increased the polydispersity index of PVPSeNPs, but it did not affect the size distribution of PS-stabilized NPs.

The zeta potential of a nanoparticle is the measure of its surface charge density; it affects the stability of the NPs, but it also determines the way in which they interact with biological systems. Surface modification, which can be measured by the change in zeta potential, can affect solubility, mucus transport, cellular transport, etc. Negative values of zeta potential are also an indicator of lower cytotoxicity [51].

The zeta potential of the NPs ranged from −5.9 mV (PVPSeNPs) to −33.0 mV (SeNPs coated with PS-PSSeNP) (Table 1) and it was significantly affected by OPE (−10.8 mV), indicating that the modification of the nanoparticle surface with OPE polyphenols was achieved. On the other hand, the zeta potential of PSSeNPs (−33 mV) was unaffected by functionalization with OPE. Absolute values of zeta potential of 30–40 mV (as in the case of PS-stabilized NPs) indicate particularly good stability. However, adequate stabilization can also be achieved with low absolute values of zeta potential by using steric stabilizers, such as PVP.

The stability of the NPs was investigated in two media (deionized water and the buffer solution used for the biocompatibility studies) within a period of 48 h. As presented in Figure 3, the observed changes in the average diameter and zeta potential were not significant, indicating good stability of the investigated nanosystems.

### 2.3. Bioaccessibility of SeNPs

The biogenic synthesis of SeNPs has been investigated previously and, in most cases, biological sources were utilized as reducing agents (such as bacteria, fungi, yeasts and plants) and the obtained nanosystems were characterized in terms of the physico-chemical properties, biocompatibility, and antimicrobial and antioxidative properties [52]. However, synthesis processes focused on utilizing food-waste extracts in the production of SeNPS are scarce [53] as are data on their gastrointestinal stability/bioaccessibility (as a prerequisite to SeNPs’ efficiency as nutraceuticals).

Bioaccessibility is defined as the amount of an ingested nutraceutical that is available for absorption in the gut after digestion and, therefore, reflects the gastrointestinal stability of the investigated compound. It is an important characteristic for both therapeutic and diagnostic applications of NPs, as well as for inadvertent exposure, due to its influence on the toxicity of nanomaterials. This was investigated via an in vitro approach whereby SeNPs were submitted to a simulation of gastrointestinal digestion, which has been proven by other authors to be applicable in nanoformulations [54,55].

As presented in Table 2, the gastrointestinal stability of the investigated SeNPs ranged from 33.57% (PVPSeNPs) to 56.93% (fPVPSeNPs), and it was significantly increased by the functionalization of the NPs’ surface with OPE—by 69.59% in PVPSeNPs and by 20.77% in PSSeNPs. The more pronounced effect visible, in the case of PVPSeNPs, indicates more intensive surface modification by OPE in comparison to PSSeNP, which is consistent with the observed effects of functionalization on the zeta potential of SeNPs. The observed decrease in bioaccessibility might be explained by the fact that under gastrointestinal conditions, SeNPs release Se, which is subsequently oxidized within the gastrointestinal lumen to Se^4+^, as recently shown by Chen and co-authors (2022) [55]. This means that, when applied in the form of SeNPs, selenium will be absorbed as both SeNPs and selenite. According to the conclusions of Wiecinsky and co-authors (2009) [54], SeNPs are particularly sensitive to low gastric pH, while the effects of intestinal pH/enzymes are much less pronounced.

### 2.4. Biocompatibility of SeNPs

**Figure 4:**
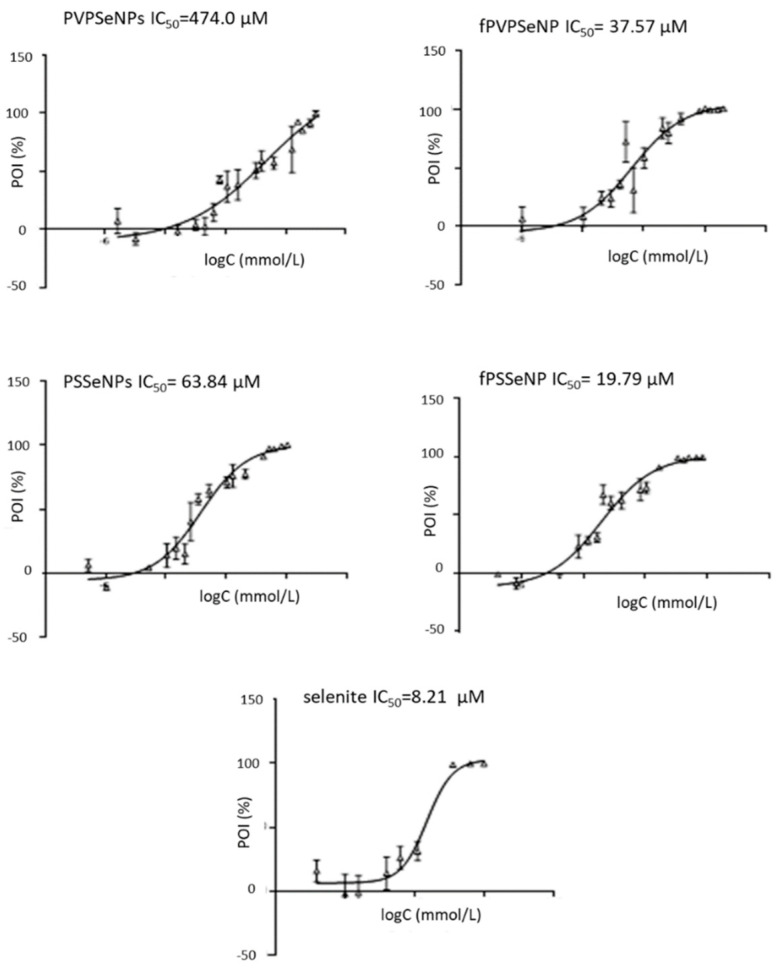
Cytotoxicity of different concentrations of selenium nanoparticles and sodium selenite in HepG2 cell lines. POI-percentage of inhibition; PVPSeNPs—selenium nanoparticles coated with polyvinylpyrrolidone; fPVPSeNPs—selenium nanoparticles coated with polyvinylpyrrolidone and functionalized with olive pomace extract; PSSeNPs—selenium nanoparticles coated with polysorbate; fPSSeNPs—selenium nanoparticles coated with polysorbate and functionalized with olive pomace extract.

**Figure 5:**
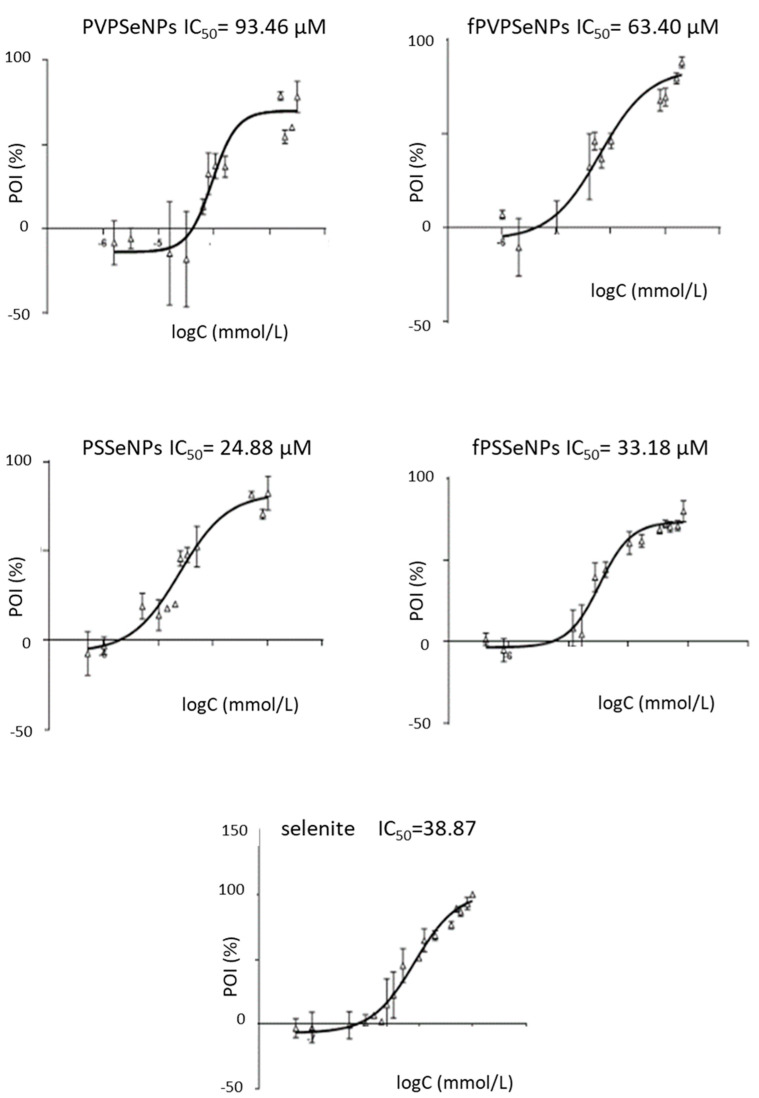
Cytotoxicity of different concentrations of selenium nanoparticles and sodium selenite on Caco2 cells. POI-percentage of inhibition; PVPSeNPs—selenium nanoparticles coated with polyvinylpyrrolidone; fPVPSeNPs—selenium nanoparticles coated with polyvinylpyrrolidone and functionalized with olive pomace extract; PSSeNPs—selenium nanoparticles coated with polysorbate; fPSSeNPs—selenium nanoparticles coated with polysorbate and functionalized with olive pomace extract.

Biocompatibility studies were conducted using two immortalized cell lines—HepG2 and Caco2. Those established cell culture models have been previously used to provide useful information on toxicity and mechanisms that could help to better inform safety assessments of diverse types of NP. The biocompatibility of different SeNPs was compared via calculation of the IC_50_ values obtained using the MTT-test, as presented in Figure 4 and Figure 5.

The results obtained in the HepG2 cells showed that all SeNPs had significantly lower toxicity in comparison to inorganic selenite (19.79–474.00 vs. 8.21 µM). Generally, PVP-coated NPs showed higher IC_50_ values, indicating lower toxicity in comparison to PS-coated systems, which is in agreement with one of our previous studies [21]. This could be due to the significantly lower average diameter of PS-coated NPs. As mentioned previously, nanoparticle size is one of the key factors that determine the rate of cellular internalization; therefore, it can be assumed that a smaller size led to their stronger penetration inside the cells and to the reduction in cell viability [49]. Additionally, the chemical nature of surfactants makes them potentially deleterious to cell membranes, which, in turn, could be the cause of the strong cytotoxic effect of PSSeNPs. The functionalization of the NP surface with OPE (in both, PVP- and PS-coated NPs) resulted with lower IC_50_ values, indicating lower biocompatibility and potential anticancerogenic effects. The observed effects could be due to the lower average diameter of functionalized NPs; namely, the toxicity of NPs is also inversely correlated with the diameter of the particle. Smaller NPs (1–100 nm) show increased toxicity because they are comparable to protein globules (2–10 nm), to the diameter of a DNA helix (2 nm), and to the thickness of cell membranes (10 nm). This allows them to easily penetrate cells (and cell organelles). NPs < 10 mm are particularly toxic since they can enter the cell nucleus [56]. Additionally, functionalization of the surface of NPs with OPE could alter their interactions with cell structures and result in the synergistic effects of SeNPs and OPE polyphenols, which are known to exert anticancer effects against Caco2 and HepG2 cells [57]; namely, as reported previously, OPE contained a significant amount of hydroxytyrosol, tyrosol and oleuropein and showed strong antiradical activity [39].

The results obtained on Caco-2 cells partially differed from those obtained on HepG2 cells. Again, PVPSeNPs showed lower toxicity in comparison to PSSeNPs; however, the effect on functionalization with OPE was not as pronounced as in the HepG2 cell line. Moreover, in the case of PS-coated NPs, functionalization increased the IC_50_ values, resulting in decreased toxicity. This might indicate that inorganic selenite was less toxic to Caco2 cells and the obtained IC_50_ values were comparable to those obtained for fPSSeNPs. Generally, Caco-2 cells were less sensitive to SeNPs in comparison to HepG2 cells, which is in line with other available data [58].

The results of this study suggest that the HepG2 and Caco2 cells in culture respond to SeNPs, making them suitable in vitro models for investigating their cytotoxicity. The observed differences in cellular responses indicate differences in the mechanisms of toxicity induced by SeNPs, depending on the types of cell used.

## 3. Materials and Methods

### 3.1. Preparation of OPE

The OPE was prepared according to a previously optimized procedure with some modifications [59]. Briefly, dry, milled and sieved olive pomace was extracted using 60% (*v*/*v*) ethanol in deionized water via constant shaking at 70 °C for 2 h. The extract was filtrated, lyophilized and stored at −20 °C for future analysis. For SeNP synthesis, the dry extract was dissolved in deionized water to give 1% (*m*/*v*) solution and filtered through 0.45 μm polyethersulfone (PES) HPLC syringe filters (Macherey-Nagel, Düren, Germany).

### 3.2. Optimization of Synthesis and Purification of SeNPs

The preparation of SeNPs was based on previously reported procedures [22,60,61,62] with additional modifications. Briefly, sodium selenite (0.1 M) was added gradually (1 drop every 2 s) to a mixture containing water, stabilization agent PVP or PS, reducing agent (L-ascorbic acid) and, facultatively, olive pomace extract for functionalized NPs, as presented in Table 3. The synthesis was conducted using a magnetic stirrer under constant stirring conditions at room temperature for 20 min.

After the synthesis, for the purpose of purification and the removal of the reactants remaining in reaction mixture, the samples were transferred to Falcon cuvettes and centrifuged (procedure A), centrifuged in Amicon*^®^* cuvettes (procedure B) or dialyzed (procedure C).

Procedure A was conducted in Falcon cuvettes at 10,000 g for 15 min. The supernatants were removed and the NPs were resuspended in 10 mL of deionized water and centrifugated again under identical conditions. For procedure B, Amicon*^®^* cuvettes (Amicon^®^ Ultra-15 10K, Merck, Kenilworth, NJ, USA) were used. A total of 15 mL of the reaction mixture was added to the upper chamber of the cuvette and the samples were centrifuged at 4000× *g* for 20 min. The sample remaining in the upper chamber was resuspended in 10 mL of deionized water and centrifugated again. For procedure C, 30 mL of the reaction mixture was transferred into dialysis bags (dialysis tubing cellulose, Sigma-Aldrich, St. Louis, MO, USA; MWC 14,000 Da) and dialyzed. To optimize the dialysis process and ensure complete purification of the SeNPs, the ionized water (outer phase) was changed several times during dialysis until its conductivity (SevenMulti, Mettler Toledo, Schwerzenbach, Switzerland) reached the conductivity of pure deionized water (0.99 μS/cm), indicating the end of the dialysis process.

The samples obtained after centrifugation/dialysis were collected, evaporated to dryness using a rotavapor and stored for future analyses.

### 3.3. Characterization and Stability of SeNPs

Prior to the analysis, the dry SeNPs were dissolved in an adequate amount of deionized water. The relative content of Se in the obtained solutions was determined spectrophotometrically as suggested by other authors [63]. The absorption wavelength (350 nm) was determined based on the preliminary analysis of the absorption spectra of the obtained SeNPs where all the investigated mixtures showed similar absorption maxima (350 ± 12 nm).

The visualization of SeNPs was performed using transmission electron microscopy (TEM). The samples were prepared by pipetting a drop of the SeNP solution on top of the Formvar*^®^*-coated copper grid (SPI Supplies, West Chester, PA, USA) and leaving it to dry for 24 h at RT. The microscope (TEM 902A, Carl Zeiss Meditec Ag, Jena, Germany) was operated in bright-field mode with an acceleration voltage of 80 kV. The images were obtained using a Canon PowerShot S50 camera (Canon, Tokyo, Japan).

Dynamic light scattering (DLS) and electrophoretic light scattering (ELS) were used to measure the hydrodynamic diameters (dH) and zeta potentials (ζ) of the SeNPs, respectively. The measurements were performed using a Zetasizer Nano ZS (Malvern Instruments, Malvern, UK) equipped with 532 nm green laser. The intensity of the scattered light was detected at an angle of 173°. All measurements were performed at room temperature. Zetasizer software 6.32 (Malvern Instruments, Malvern, UK) was used for data analysis. The hydrodynamic diameter distributions were obtained using the size–volume distribution function and the dH is reported as an average value of 10 measurements. The ζ potential was calculated using the Henry equation with Smoluchowski approximation and is reported as an average of 5 measurements. As indicators of nanoparticle stability, changes in the hydrodynamic diameter, size distribution and surface charge were investigated. The measurements were performed in two selected media—ultrapure water (UPW) and phosphate-buffered saline (PBS)—during three selected timeframes: 0, 4 and 24 h.

### 3.4. Determination of TEAC

The scavenging activity of the SeNPs against the 2,2’-azino-bis(3-ethylbenzothiazoline-6-sulfonic acid radical cation (ABTS+) was determined sing a colorimetric assay originally described by Re and co-authors (1999) [64]. An ABTS+· solution was prepared using 7 mmol/L aqueous ABTS solution reacting with 2.45 mmol/L potassium persulfate solution in the dark at 4 °C for 12 h. After the reaction, the ABTS+· solution was diluted with distilled water to give an absorbance of 0.70 ± 0.02 at 732 nm (Victor X3, PerkinElmer, Waltham, MA, USA). A total of 20 μL of the sample/Trolox*^®^* standard/blank was pipetted into a microplate well in triplicate and 200 μL of diluted ABTS+· was added. The absorbance was measured at 750 nm after 90 s of incubation at 30 °C. The percentage of quenching the absorbance was calculated according to Equation (1):

∆A = (A0min − A3min)/A0min × 100(1)

A calibration curve was generated by plotting different Trolox*^®^* concentrations against their respective absorbance-quenching percentages. The antiradical efficiency was expressed as mg of Trolox*^®^* equivalents (mg/g TE).

### 3.5. Determination of Gastrointestinal Bioaccessibility of SeNPs

The bioaccessible fractions of the SeNPs were obtained via in vitro static simulation of gastrointestinal digestion according to the standardized protocol described by Brodkorb and co-workers (2019) [65]. Samples were initially incubated in simulated gastric fluid (SGF/pepsin) to simulate gastric conditions (37 °C for 2 h in a water bath (Büchi B-490, Flawil, Switzerland)) with uniform shaking at 110 rpm. The simulated intestinal fluid (SIF/bile salt/pancreatin) was added to the samples and the reaction mixtures were incubated under the same conditions for a subsequent 2 h. Then, the incubation samples were put on ice for 10 min and filtrated through polypropylene hydrophilic membranes (pore diameter 20 µm) to obtain clear filtrates suitable for the spectrophotometric determination of SeNPs (as described in Section 2.3). The results are expressed as the relative amounts of SeNPs (in relation to the initial concentration of NPs prior to the simulation of the gastrointestinal process).

### 3.6. Determination of Biocompatibility of SeNPs

The HepG2 cells were cultured in essential growth medium (EMEM) supplemented with 10% fetal bovine serum (FBS) (Cat. No. FBS-HI-11A; Capricorn Scientific, Ebsdorfergrund, Germany). The caco-2 cells were cultured in Dulbecco’s Modified Eagle Medium (DMEM) supplemented with 20% FBS. Both media were additionally supplemented with 1% antibiotic/antimycotic solution (Sigma-Aldrich, St. Louis, MO, USA), 1% MEM Nonessential Amino Acids (Capricorn Scientific GmbH, Ebsdorfergrund, Germany) and 4 mM L-glutamine (Sigma-Aldrich, St. Louis, MO, USA). The cells were kept in a cell culture incubator at 37 °C with 5% CO_2_ and at >80% humidity. The medium was changed every few days until the cells reached 80% confluence. Before each experiment, the cells were treated with 1× Trypsin (2.5% in HBSS w/o Ca, Mg, Lonza, Basel, Switzerland) diluted in ethylenediaminetetraacetic acid solution (EDTA) (Sigma-Aldrich, St. Louis, MO, USA) to allow the cells to detach from flask surface. The number of cells was estimated using a hemocytometer (Neubauer, Germany).

For the determination of cell viability, 20,000 cells per well were seeded in a 96-well plate and incubated for 48 h. Fresh medium was added, and the cells were incubated with NPs for 24 h. The cells were washed two times with PBS followed by the addition of a 3-(4,5-Dimethylthiazol-2-yl)-2,5-diphenyltetrazolium bromide (MTT) reagent (Carbosynth Limited, Compton, UK). After 3 h of incubation, dimethyl sulfoxide (DMSO) was added into every well and the plate was put on a shaker for 45 min. The absorbance was measured at 530 nm using a multilabel plate reader (Victor X3, PerkinElmer, Waltham, MA, USA).

### 3.7. Statistical Analysis

All experiments were run in quadruplicate unless otherwise stated. The data from the bioaccessibility and antioxidant assays were statistically tested via one-way analysis of variance (ANOVA), followed by Tukey’s multiple comparisons test. The results were expressed as the average value and standard deviation. *p* < 0.05 was considered statistically significant unless otherwise noted. For biocompatibility assays, the data analysis included a calculation of the percentage of inhibition (POI) for every tested concentration of SeNPs, which were then used to calculate the 50% inhibitory concentration (IC_50_) using nonlinear regression. All the results were analyzed using GraphPad*^®^* Prism 6 Software (San Diego, CA, USA).

## 4. Conclusions

Olive pomace extract was successfully applied for the functionalization of PVP- or PS-coated SeNPs, showing positive effects on the yields of the proposed synthesis protocols. The green synthesis process—which utilized food-grade reactants and bioactive compounds extracted using sustainable procedures from food waste—was optimized in terms of obtaining stable spherical nanosystems characterized by unimodal particle-size distribution and average diameters suitable for peroral application (56.0–181.7 nm). The functionalization with OPE significantly reduced NP size and reduced the zeta potential. The obtained NPs were relatively unstable under gastrointestinal conditions; the average bioaccessibility ranged from 33.57 to 56.93% and was positively affected by functionalization with OPE. The biocompatibility studies confirmed the lower toxicity of most of the SeNPs in comparison to sodium selenite and showed that the functionalization of NPs with OPE significantly affects the biocompatibility of derived nanosystems, depending on the different cell lines.

## Figures and Tables

**Table 1:** Average yield, diameter, zeta potential and polydispersity index of SeNPs.

Sample	Yield (ppm)	Average Diameter (nm)	Zeta Potential (mV)	Polydispersity Index	pH
PVPSeNPs	3.27 ± 0.12 ^a^	181.7 ± 0.58 ^a^	−5.9 ± 0.48 ^a^	0.11 ± 0.02 ^a^	8.16
PSSeNPs	1.62 ± 0.09 ^b^	74.9 ± 0.29 ^b^	−33.0 ± 1.22 ^b^	0.12 ± 0.01 ^a^	7.94
fPVPSeNPs	4.38 ± 0.24 ^c^	107.6 ± 0.36 ^c^	−10.3 ± 0.99 ^c^	0.18 ± 0.03 ^b^	7.88
fPSSeNPs	2.34 ± 0.12 ^d^	56.0 ± 0.45 ^d^	−30.0 ± 1.05 ^d^	0.12 ± 0.01 ^a^	7.61

PVPSeNPs—selenium nanoparticles coated with polyvinylpyrrolidone; fPVPSeNPs—selenium nanoparticles coated with polyvinylpyrrolidone and functionalized with olive pomace extract; PSSeNPs—selenium nanoparticles coated with polysorbate; fPSSeNPs—selenium nanoparticles coated with polysorbate and functionalized with olive pomace extract. Data in the same column marked by different letters are significantly different (*p* < 0.05).

**Table 2:** Gastrointestinal stability and bioaccessibility of SeNPs.

	Nanoselenium Content (ppm)		Intestinal Bioaccessibility (%)
	Initial	Intestinal	
PVPSeNPs	44.60 ± 0.47 ^a^	14.97 ± 1.28 ^a^	33.57 ± 2.87 ^a^
PSSeNPs	80.94 ± 0.87 ^b^	32.82 ± 1.78 ^b^	40.54 ± 2.20 ^b^
fPVPSeNPs	33.13 ± 0.82 ^c^	18.86 ± 0.51 ^c^	56.93 ± 1.55 ^c^
fPSSeNPs	31.59 ± 0.96 ^c^	15.47 ± 1.00 ^a^	48.96 ± 3.15 ^d^

PVPSeNPs—selenium nanoparticles coated with polyvinylpyrrolidone; fPVPSeNPs—selenium nanoparticles coated with polyvinylpyrrolidone and functionalized with olive pomace extract; PSSeNPs—selenium nanoparticles coated with polysorbate; fPSSeNPs—selenium nanoparticles coated with polysorbate and functionalized with olive pomace extract). Data in the same column marked by different letters are significantly different (*p* < 0.05).

**Table 3:** Composition of reaction mixtures used for synthesis of SeNPs.

Sample	ASC (0.1 M)	PVP (1%)	PS	OPE (1%)	Na_2_SeO_3_ (0.1M)	Water	Total
	mL						
PVPSeNPs	3.3	3	0	0	0.33	23.37	30
PSSeNPs	1.7	0	0.08	0	0.35	27.87	30
fPVSeNPs	3.3	3	0	5	0.33	18.37	30
fPSSeNPs	1.7	0	0.08	5	0.35	22.87	30

ASC-L—ascorbic acid; PVP—polyvinylpyrrolidone; PS—Polysorbate Tween^®^20; OPE—olive pomace extract.

## Data Availability

Not applicable.
